# The P2X7 receptor regulates cell survival, migration and invasion of pancreatic ductal adenocarcinoma cells

**DOI:** 10.1186/s12943-015-0472-4

**Published:** 2015-11-25

**Authors:** Andrea Giannuzzo, Stine Falsig Pedersen, Ivana Novak

**Affiliations:** Department of Biology, Section of Cell Biology and Physiology, University of Copenhagen, August Krogh Building, Universitetsparken 13, DK-2100 Copenhagen, Denmark

**Keywords:** P2X7, Purinergic signaling, Pancreatic ductal adenocarcinoma, Pancreatic cancer, AZ10606120, KN-62, A438079, Cell proliferation, P2Y receptors

## Abstract

**Background:**

Pancreatic ductal adenocarcinoma (PDAC) is presently one of the cancers with the worst survival rates and least effective treatments. Moreover, total deaths due to PDAC are predicted to increase in the next 15 years. Therefore, novel insights into basic mechanism of PDAC development and therapies are needed. PDAC is characterized by a complex microenvironment, in which cancer and stromal cells release different molecules, such as ATP. ATP can be transported and/or exocytosed from active cancer cells and released from dying cells in the necrotic core of the cancer. We hypothesized that one of the ATP receptors, the P2X7 receptor (P2X7R) could be an important player in PDAC behaviour.

**Methods:**

We determined the expression (real time PCR and Western blot) and localization (immunofluorescence) of P2X7R in human PDAC cell lines (AsPC-1, BxPC-3, Capan-1, MiaPaCa-2, Panc-1) and a “normal” human pancreatic duct epithelial cell line (HPDE). The function of P2X7R in proliferation (BrdU assay), migration (wound assay) and invasion (Boyden chamber with matrigel) was characterized. Furthermore, we studied P2X7R-dependent pore formation (YoPro-1 assay) and cell death (caspase and annexin V / propidium iodide assays).

**Results:**

We found higher expression of P2X7R protein in PDAC compared to HPDE cells. P2X7R had notable disparate effects on PDAC survival. Firstly, high concentrations of ATP or the specific P2X7R agonist, BzATP, had cytotoxic effects in all cell lines, and cell death was mediated by necrosis. Moreover, the P2X7R–pore antagonist, A438079, prevented ATP-induced pore formation and cell death. Second, in basal conditions and with low concentrations of ATP/BzATP, the P2X7R allosteric inhibitor AZ10606120 reduced proliferation in all PDAC cell lines. P2X7R also affected other key characteristics of cancer cell behavior. AZ10606120 reduced cell migration and invasion in PDAC cell lines compared to that of untreated/vehicle-treated control cells, and stimulation with sub-millimolar concentrations of ATP or BzATP substantially increased cell invasion.

**Conclusions:**

PDAC cell lines overexpress P2X7R and the receptor plays crucial roles in cell survival, migration and invasion. Therefore, we propose that drugs targeting P2X7R could be exploited in therapy of pancreatic cancer.

**Electronic supplementary material:**

The online version of this article (doi:10.1186/s12943-015-0472-4) contains supplementary material, which is available to authorized users.

## Background

Pancreatic Ductal Adenocarcinoma (PDAC) is an aggressive and devastating disease [1]. The estimated 5-year survival rate is less than 5 %, even in regions of the world with the best healthcare, and incidence is expected to rise with ageing population [2]. In USA, total deaths due to pancreatic cancer (where PDAC constitute about 90 % of these cancers), are projected to increase dramatically and become the second leading cause of cancer-related deaths before 2030 [3]. Due to redundancy of pancreatic function, the disease is detected relatively late, when the local tumor has advanced or the disease has disseminated. Most conventional chemotherapies fail to give substantial responses in PDAC and one of the important factors may be the unusual and impermeable tumor microenvironment (TME). PDAC is a solid tumor rich in stromal cells and exhibiting marked desmoplasia, stiffness and poor vascularization [4]. The cellular compartment of TME includes, apart from cancer cells, pancreatic stellate cells (PSCs), cancer associated fibroblasts, various immune cells, endothelial cells and pericytes. The acellular component includes, extracellular matrix (ECM) components and matrix-degrading enzymes, as well as a number of growth factors and cytokines [4], autocrine/paracrine factors, metabolites and ions. In the complex TME, cancer and stromal cells release a variety of molecules that can support tumor proliferation, migration, invasion and immune system escape [5]. One relevant and multifunctional candidate is ATP, which can be released from metabolically active cancer cells and other cells to the extracellular space via plasma membrane transport systems or by exocytosis, and from dying cells in the tumor necrotic area [6]. It is difficult to detect intra-tumor ATP levels due to up-regulated ecto-nucleotidases and hydrolysis of ATP to adenosine [7]. Using plasma membrane luciferase and bioluminescence detection, it has been possible to detect extracellular ATP at hundreds micromolar concentration in tumor interstitium in melanoma and ovarian cancer [8]. However, methods to monitor ATP concentration gradients across tissues or tumors are not yet available.

ATP and other extracellular nucleotides activate two families of receptors: P2X receptors, a family of ligand-gated receptor channels; and P2Y receptors, G protein-coupled receptors [9]. One of the most remarkable purinergic receptors is the P2X7 receptor, because it has a crucial role in several physiological and pathophysiological processes and because the receptor is highly polymorphic, and single nucleotide polymorphism (SNPs) are associated with several diseases including central nervous system diseases, pain, osteoporosis, cancer and inflammation [10]. There are nine different human splice variants - named P2X7A–J (P2X7B, P2X7C, P2X7E, P2X7G and P2X7J lack of C-terminal) [10], and isoforms A and B are the most studied. P2X7 receptor (P2X7R) is a two membrane-spanning domain protein that forms trimers and at submillimolar extracellular ATP concentrations behaves as a cation channel and elicits a number of cellular responses. An extensively documented and well established role of the receptor is in cell death. At millimolar ATP concentrations P2X7R mediates plasma membrane permeabilization, due to formation of pores permeable to large molecules (up to 900 Da) [11, 12], and this leads to apoptotic/necrotic events [13, 14]. Recently, several reports showed a novel characteristic of P2X7R, namely that it can increase cell proliferation [15, 16]. This effect may be elicited at basal or low ATP concentrations [17, 18] and/or depends on isoforms expression [19].

The P2X7 receptor is over-expressed in many cancer types [20–22], and potentially the unusual double role of P2X7R in cancer cell proliferation and cell death and modulation by immune system could find applications in cancer therapy. Two hypotheses regarding the roles of P2X7R are proposed: either the receptor can be considered as an anti-tumor protein inducing cell death in cancer cells [23–25]; or the receptor is a pro-cancerous protein increasing tumor growth and invasion [26, 27]. Given that the TME is likely to exhibit ATP concentration gradients, the receptor could serve different functions in different locations in the tumor [6].

In pancreas, P2X7R is expressed in rodent duct cells (but not acinar cells) and in human PDAC cells grown as epithelial monolayers, and in both the receptor has a role in calcium signaling and regulation of ion transport and secretion [28–30]. Moreover, P2X7R is expressed in pancreatic stellate cells (PSCs) [31], which are the major contributors to the abundant stromal/desmoplastic reaction that characterizes pancreatic cancer [32]. The role of P2X7R in PSCs is to support proliferation and/or cell death depending on extracellular ATP concentrations [17]. One study utilizing patient tissues reported that there was a tendencial increase of P2X7R protein level in chronic pancreatitis and pancreatic cancer compared to normal tissues [33].

In light of the fact that this receptor is important for cell survival and may be overexpressed in pancreatic cancer, we need to understand its role in PDAC behavior. The aim of this study was to use a simple *in vitro* cell model to detect the expression of P2X7R in PDAC cell lines and to clarify whether it affects PDAC behavior such as cell proliferation, cell death, migration and invasion. Knowledge gained from this study can form the basis for more advanced drug testing in *in vivo* pancreas cancer models.

## Results

### Expression and localization of P2X7 receptor in PDAC and control human pancreatic duct cell lines

Five PDAC cell lines were used: AsPC-1, BxPC-3, Capan-1, MiaPaCa-2 and Panc-1. They are genotypically and phenotypically heterogeneous and they are representative of different stages of pancreatic cancer. For example, Panc-1 is derived from epithelioid pancreatic carcinoma, MiaPaCa-2 is a poorly differentiated cell line [34], Capan-1 is a well differentiated cell line derived from liver metastasis [35], and AsPC-1 is a poorly differentiated cell line derived from nude mouse xenografts initiated with cells from the ascites of a patient with pancreatic cancer [36]. All cell lines have mutations in *TP53* and *KRAS* genes, except for BxPC-3 which has wild type *KRAS*. HPDE, human “normal” pancreatic duct epithelial cell line, transformed using HPV16-E6E7 [37] was used as control.

**Fig. 1:**
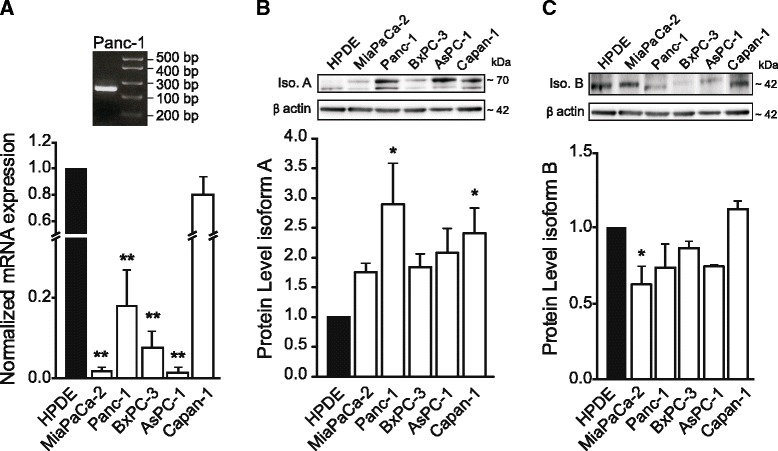
Expression of P2X7R in PDACs and HPDE cells. **a**. Real time PCR and RT-PCR analysis of P2X7R expression in HPDE and PDAC cells. Insert shows a representative gel of P2X7R mRNA (284 bp) in Panc-1. The data were normalized with respect to the three housekeeping genes: *β-actin*, β glucuronidase (*GUSB*) and glutaminyl-tRNA synthetase (*QAR*S) and expression in HPDE was set to 1. The bargraph shows data for four experiments (mean ± SEM). The relative amount of mRNA was calculated from a standard curve run on each plate. **b**. Western blot band at around 70 kDa corresponding to the isoform A [PR: Q99572-1], as shown on the insert. Loading control was β-actin [PR: P60709] detected at 42 kDa. All lanes were loaded with 50 μg of proteins. **c**. Western blot band at around 42 kDa most likely corresponds to the isoform B [PR: Q99572-2]. The results were normalized with β-actin that was used as a loading control. The graphs for the two isoforms show data of three experiments (mean ± SEM). Significant differences of expression in PDAC in comparison to HPDE cells P < 0.05 (*) and P < 0.001 (**) are indicated

The primer set for P2X7 detection (284 bp) (Table 1) was designed such that the expression of the isoforms A and B (and also G, H, J, D) would be detected in a real time PCR. The forward primer would recognize the junction between exon 3 and 4, and the reverse primer the junction between the exon 6 and 7. The P2X7R expression among the different cell lines was normalized with respect to HPDE, after normalization of each cell line to average expression of *QARS*, *GUSZ* and *β-actin* housekeeping genes. Figure 1a shows that compared to HPDE cells, there was a significant down-regulation of P2X7R transcripts in all the PDAC cell lines, except for Capan-1 cells. In addition to P2X7R, pancreatic duct cells also express a number of other P2X and P2Y receptors and additional data for the key receptors transcripts are given in Additional file 1: Figure S1 and primers are in Additional file 2: Table S1.

**Table 1:** Primers used for RT-PCR and Real Time PCR on PDACs and HPDE

Primers	Accession numbers	Sequence	Product length
P2X7 FW	[GenBank: GQ180122.1]	CGGTTGTGTCCCGAGTATCC	284 bp
P2X7 RW		CCTGGCAGGATGTTTCTCGT	
β actin FW	[GenBank: BC014861.1]	GTGACATTAAGGAGAAGCTGTGC	300 bp
β actin RW		CAATGCCAGGGTACATGGTG	
GUSB FW	[GenBank: M15182.1]	ACTGACACCTCCAAGTATCCCA	200 bp
GUSB RW		AACAGGTTACTGCCCTTGACAG	
QARS FW	[GenBank: BC000394.2]	CCTCTATGAGCGACTATTCCAGC	200 bp
QARS RW		GATGGCTGTCTGGATCCACG

Protein expression of the full length P2X7R A isoform and the C-terminus truncated B isoform was determined using Western blot and immunolocalization (Fig. 1b-c). Figure 1b shows two bands, often seen by other researchers, and the lower band may correspond to the isoform H. The band at 70 kDa, corresponding to the isoform A, appears more abundant in all PDAC cell lines compared to control HPDE cells, but significant increase is detected only for Capan-1 and Panc-1. Figure 1c shows that there was a slightly reduced, but not significant, expression of the 42 kDa band that possibly corresponds to the isoform B in AsPC-1, BxPC-3 and Panc-1; and there is significantly lower expression in MiaPaCa-2 compared to HPDE cells.

**Fig. 2:**
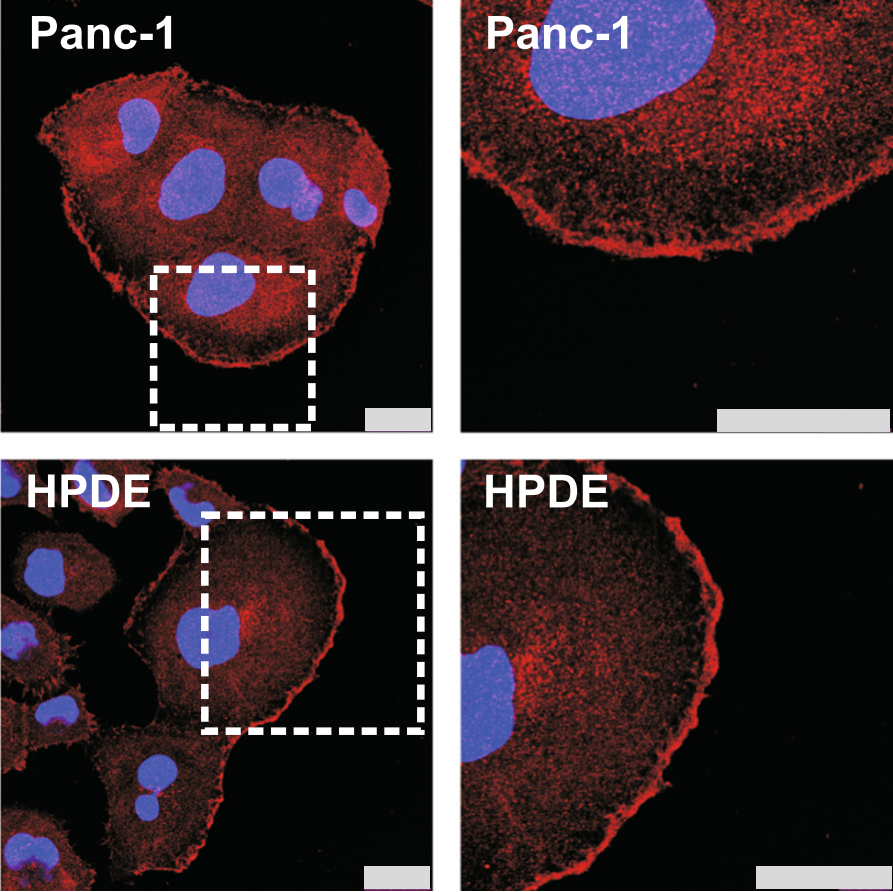
Immunolocalization of P2X7R in PDACs and HPDE cells. HPDE and Panc-1 cells were grown on coverslips. P2X7R was stained with a polyclonal antibody against the extracellular domain and Alexa Fluor 568 (red) and DAPI was used to stain the nucleus (blue). All bars are 25 μm

Localization of P2X7R was detected by immunofluorescence and confocal microscopy and the results are shown in Fig. 2. The immunofluorescence was observed in HPDE and Panc-1 cells and the immunofluorescence signal resulting from the polyclonal antibody recognizing the extracellular loop, hence the A and B and potentially other isoforms, was localized to the plasma membrane, and weakly also in the cytoplasmic compartment (Fig. 2).

### P2X7R affects cell proliferation and cell death

**Fig. 3:**
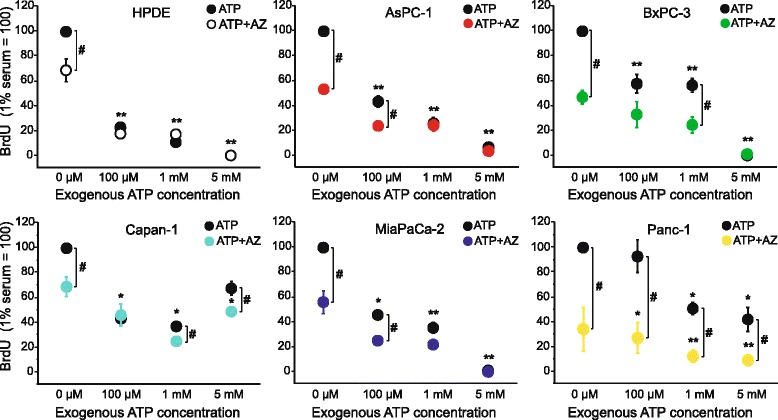
Effect of ATP and AZ10606120 on BrdU incorporation in PDACs and HPDE cells. Data for each cell line are given in one panel and filled black symbols show the effect of different concentrations of added (exogenous) ATP (100 μM, 1 mM and 5 mM), or control (no exogenous ATP added), on BrdU incorporation in all cell lines after 60 h. The colored symbols show the effect of ATP and control in combination with the allosteric inhibitor AZ10606120 (10 μM), which was added 1 h before ATP. The results were normalized to 1 % serum control (100). The graphs show data from three to six experiments (mean ± SEM); where each run was carried out in triplicates. Significant differences P < 0.05 (*, #) and P < 0.001 (**) from the respective 1 % serum control (*, **) and with/without inhibitor (#) are indicated

In the following series of experiments we investigated the effect of a sustained activation or inhibition of P2X7 receptor on cell proliferation and/or death using BrdU assay. First, cells were incubated with different concentrations of ATP and the results for the six cell lines are shown in Fig. 3 (black symbols). Generally, with increasing ATP concentration from 100 μM to 5 mM all cell lines showed a decrease in BrdU incorporation, possibly indicating cell death (see below). Minor deviations in responses were observed in Panc-1 cells, which were relatively insensitive to 100 μM ATP, and in Capan-1 cells, which showed an increase in BrdU incorporation with 5 mM ATP, but a reduction at 1 mM. This effect in Capan-1 cells could be due to a selection of resistant cells after long incubation with a high dose of ATP, as shown in murine macrophage RAW 264.7 cells [38]*.* Lower concentrations of ATP (0.5–10 μM) had small and inconsistent effects on BrdU incorporation, showing a tendency of about 10–20 % increase in BrdU incorporation (Additional file 3: Figure S2).

**Fig. 4:**
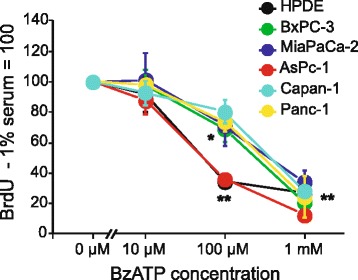
BzATP has similar effects as ATP stimulation in PDAC and HPDE. The BrdU incorporation data were normalized to 1 % serum control (100), and all cell lines were normalized with respect to the control. The black line and filled symbols show the effect of different concentration of BzATP (10 μM, 100 μM and 1 mM) on BrdU incorporation in HPDE cells after 60 h. The colour lines/symbols show the effect of BzATP on the different PDAC cell lines. The graph shows data from three to six experiments (mean ± SEM), each run was carried out in triplicates. At 100 μM BzATP, there were significant differences P < 0.05 (*) between control and treatment in BxPC-3 and P < 0.001 (**) in HPDE and AsPC-1 cells. At 1 mM BzATP there was significant inhibition P < 0.001 (**) of BrdU incorporation in all cell lines compared their respective controls

In order to verify that the above described effects of ATP were due to P2X7R, a more specific agonist, BzATP, was used and the results are shown in Fig. 4. The application of BzATP at 10 μM had no significant effects on all cell lines. With an increase of the concentration of BzATP to 100 μM, a significant reduction of BrdU incorporation was detected in HPDE, AsPC-1 (around 65 %) and BxPC-3 (around 30 %) cells. At high concentration of BzATP (1 mM), all the cell lines showed a significant decrease in BrdU incorporation (between 65 % and 85 %).

**Fig. 5:**
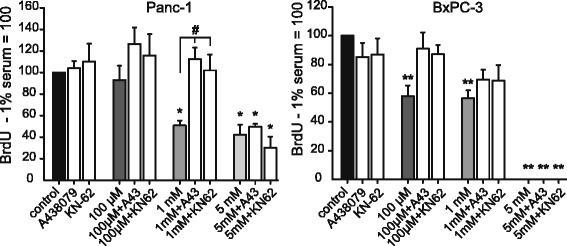
Effect of ATP and A438079, KN-62 on BrdU incorporation in Panc-1 and BxPC-3. Data show the BrdU incorporation in both cell lines after 60 h. The colored columns show the effect of different concentrations of added (exogenous) ATP (100 μM, 1 mM and 5 mM). The white columns show the effect of ATP and control (no exogenous ATP added) in combination with the two inhibitors A438079 (10 μM) or KN-62 (100 nM), which were added 1 h before ATP. The data were normalized to 1 % serum control (100). The graphs show data from three to six experiments (mean ± SEM); where each run was carried out in triplicates. Significant differences P < 0.05 (*, #) and P < 0.001 (**) from the respective 1 % serum control (*, **) and with/without inhibitor (#) are indicated

In the next series of experiments we tested the effects of P2X7R inhibitors: the allosteric inhibitor AZ10606120 (10 μM) that affects cell proliferation; the competitive antagonist A438079 (10 μM) that inhibits pore formation; and the non-competitive antagonist KN-62 (100 nM) that also inhibits CaM Kinase II [24, 27, 39–41]. Experiments where AZ10606120 was given without exogenous ATP (i.e. basal conditions) resulted in decreased BrdU incorporation in all cell lines, with the largest decrease in Panc-1 and the lowest decrease in HPDE and Capan-1 cells (Fig. 3, colored symbols). This would indicate either that the inhibitor attenuated proliferation or that it was cytotoxic and promoted cell death. We excluded the cytotoxic effect of the inhibitor with a Trypan blue assay (Additional file 4: Figure S3). Therefore, the inhibitory effect of AZ10606120 in basal conditions was most likely related to a decrease in proliferation. Furthermore, the inhibitor in combination with added ATP concentrations (0.1–1.0 mM), resulted in a further reduction of BrdU incorporation in all PDAC cell lines. Accepting that the inhibitor is not cytotoxic, we presume it reduced BrdU incorporation because it inhibited cell proliferation, as also seen in other cells [17, 27, 41]. At about 5 mM ATP effects of the inhibitor were the smallest, indicating that cell death outweighted cell proliferation. Thus Fig. 3 shows two effects on BrdU incorporation in a population of cells—increasing ATP concentration causing cell death (see below) and AZ10606120 causing a decrease in cell proliferation. Interestingly, AZ10606120 was ineffective in HPDE cells treated with 0.1–5 mM exogenous ATP, indicating that in these conditions cell death was the predominant P2X7R response in these non-cancer cells. In addition to AZ10606120, we also studied the long-term effect of two other P2X7R inhibitors, the pore-inhibitor A438079 and KN-62, and the results are shown in Fig. 5. It appears that these inhibitors improved BrdU incorporation if cells were stimulated with 0.1–1 mM ATP. Thus these inhibitors protected cells against cell death until exogenous concentrations of ATP were 5 mM.

**Fig. 6:**
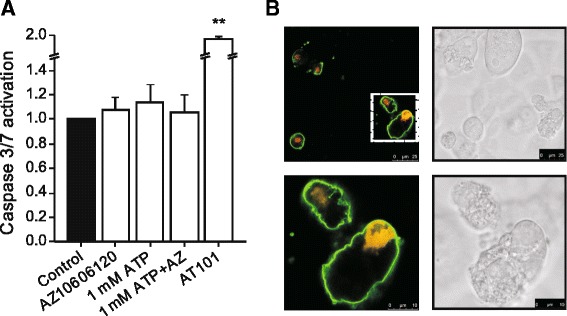
Cell death in Panc-1 cells in the presence of 1 mM ATP. **a**. Caspase 3/7 activation was measured in control cells and after 20 h incubation with 1 mM ATP, AZ10606120 (10 μM), combination of both, and AT101 (apoptosis inducer). The results were normalized to 1 % serum control (1.0). The graph shows data from four experiments (mean ± SEM); each run was carried out in duplicates. Significant difference P < 0.001 (**) from the respective 1 % serum control is indicated. **b**. Annexin V-488 (green) and propidium iodide (red) were added after 20 h incubation with 1 mM ATP in 1 % of serum. Fluorescent and transmission images were taken immediately after 10 min of incubation with the apoptosis/necrosis markers, without fixation. Images are representative from 3 independent experiments. All bars are 25 and 10 μm

The above data (Figs. 3, 4 and 5) indicated that high concentrations of ATP and BzATP could lead to cell death. We next investigated whether this was due to apoptosis or necrosis. To detect induction of apoptotic pathways, caspase 3/7 assay was used and data are shown in Fig. 6a. The administration of 1 mM ATP (20 h) had no significant effect on caspase 3/7 activation in Panc-1 cells. We moreover did not detect any effect when AZ10606120 was included alone or with 1 mM ATP. As a positive control, it is shown that apoptosis inducer AT101 (see methods) caused marked caspase activation.

In addition to caspase 3/7 assay, an Annexin V-488/propidium iodide (PI) assay was performed (Fig. 6b). Panc-1 cells were incubated with 1 mM ATP (20 h) and images in Fig. 6b show that a number of cells had red nuclei (PI), indicating that the cell membrane was permeabilized as in cells undergoing necrosis, and green Annexin V, indicating phosphatidyl serine exposure on cell surface. The transmission image also shows disrupted cells with uneven surface and nuclear swelling, also indicating a necrotic type of death.

### P2X7R inhibitors have small effect on pore formation

**Fig. 7:**
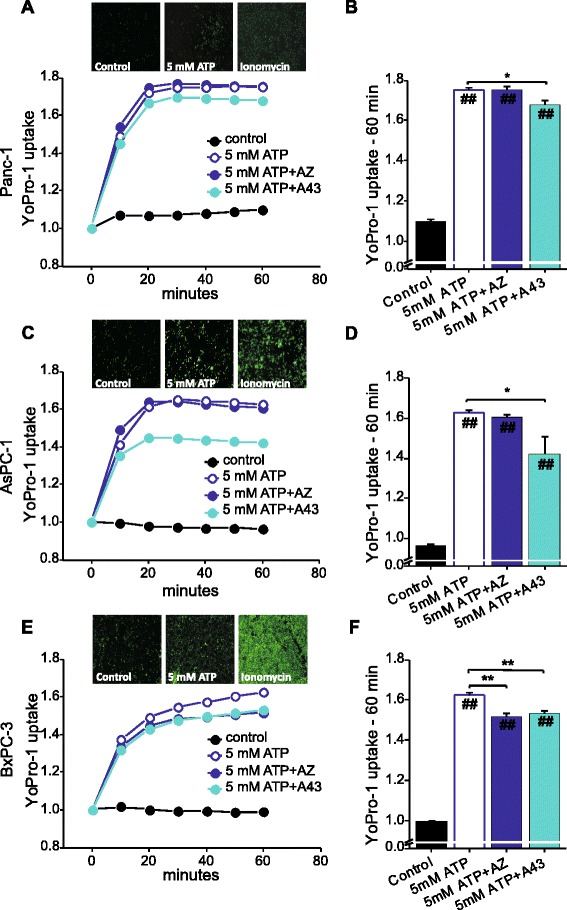
ATP stimulates pore formation. Yo-Pro-1 (2.5 μM) uptake was monitored after stimulation with 5 mM ATP alone, and in combination with a pre-inhibition (1 h) with AZ10606120 (10 μM) or A438079 (10 μM). Fluorescence of YoPro-1 (Ex. 490 nm/Em. 510 nm) was measured in Panc-1 (**a**, **b**), AsPC-1 (**c**, **d**) and BxPC-3 (**e**, **f**). **a**, **c**, **e**. Fluorescence values taken every 10 min for 60 min and normalized to the average of values before administration of ATP. Representative images of Yo-Pro-1 green fluorescence for control, 5 mM ATP and ionomycin stimulated cells (used as control to check Yo-Pro-1 efficacy) are shown as inserts for each cell lines. Note that BxPC-3 and AsPC-1 cells are more affected by ATP and ionomycin, compared to Panc-1 cells, which agrees with data in Fig. 3 and the fact that the pore inhibitor has greater effect. All bars are 100 μm. **b**, **d**, **f**. Values of normalized fluorescence at 60 min. The lines and bargraphs show data of four experiments (mean ± SEM); each run was carried out in triplicates. Significant differences P < 0.05 (*, #) and P < 0.001 (**, ##) from the respective control (#, ##) and with/without inhibitor (*, **) are indicated

One of the hallmarks of P2X7R is the ability to form pores permeable to larger molecules up to 900 Da, as shown in the above experiments. Therefore, we investigated effect of high ATP concentration (5 mM) on membrane permeabilization using YoPro-1 dye in a physiological buffer and results are shown in Fig. 7. The cell lines tested showed an increase in YoPro-1 uptake after stimulation with 5 mM ATP. The maximum YoPro-1 uptake with 5 mM ATP was detected after 20 min in AsPC-1 and Panc-1 cells, and after 50–60 min in BxPC-3 cells. We tried to inhibit P2X7R and P2X7R-mediated pore formation using two inhibitors: the allosteric inhibitor of P2X7R AZ10606120 (10 μM); and the competitive antagonist A438079 (10 μM), the latter known as the pore inhibitor. All cell lines tested showed a small but significant reduction in pore formation when A438079 was used: for YoPro-1 uptake at 60 min, this inhibition was around 6 % in BxPC-3, 13 % in AsPC-1 and 4.5 % in Panc-1 cells. A438079 protected Panc-1 cells from pore-formation in the short-term experiment (Fig. 7) and from cell death in long-term experiment where BrdU incorporation was monitored (Fig. 5). The allosteric inhibitor, AZ10606120, had no significant effect on YoPro-1 uptake in Panc-1 and AsPC-1 cells and showed a small inhibition of 6 % only in BxPC-3 cells. In an additional series of experiments we also tested 1 mM ATP on YoPro-1 uptake, but effects on pore formation were even smaller than with 5 mM ATP and these are shown in Additional file 5: Figure S4. Interestingly, although 1–5 mM ATP did not cause very pronounced pore-formation in PDAC cells in given experimental conditions and A438079 had relatively small protective effects, the inhibitor could nevertheless protected PDAC cells from cell death during long-term incubation as shown in Fig. 5.

### P2X7R increases cell migration and invasion

**Fig. 8:**
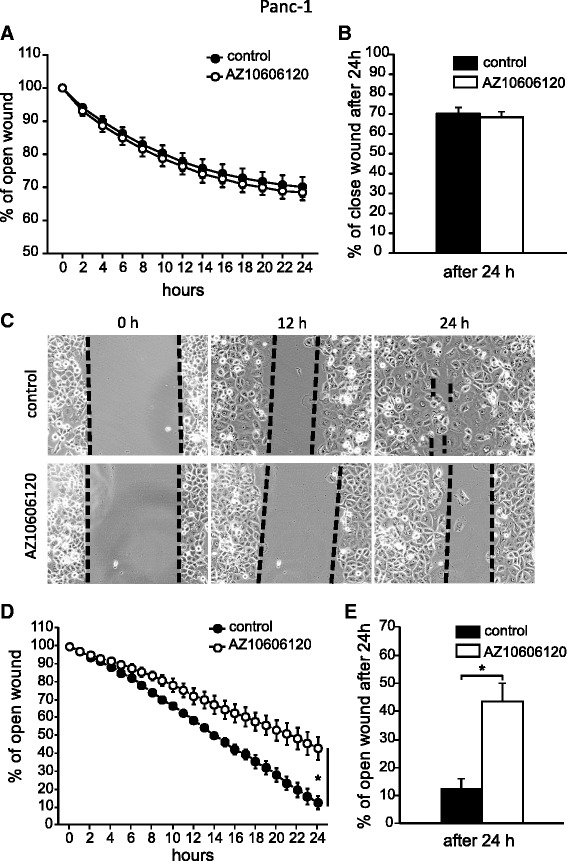
Effect of AZ10606120 on Panc-1 cells migration. Confluent Panc-1 cells, grown in 96-well plate were scratched with a 96-pin wound maker and treated with or without AZ10606120 (10 μM). **a**. The graph shows the percentage of open wound. Pictures were taken every 2 h. Images were analyzed with the IncuCyte software using the relative wound density option, which measures the density of the wound according to the density of the cell region. **b**. Percentage of wound still open after 24 h with and without inhibitor. The graphs show data of five experiments (mean ± SEM); each run was carried out in triplicates. **c**. Confluent Panc-1 cells, grown in the two chambers of an Ibidi silicon insert, were treated with or without AZ10606120 (10 μM). Phase contrast pictures were taken every hour and representative original images are shown at 0, 12 and 24 h after insert removal. **d**. Percentage of wound still open every hour with and without AZ10606120 (10 μM). Significant difference P < 0.05 (*) between control and inhibitor (**d**) is calculated on the slope of the curves. **e**. Percentage of wound still open after 24 h with and without inhibitor. In each picture the area was measured and normalized with the respective one at 0 h. In every experiment images were taken and area measured from three to seven fields. The graphs show data from three to five experiments (mean ± SEM). Significant difference calculated for wound open after 24 h

In order to determine effect of P2X7R on cell migration, a scratch assay was used and cell proliferation was inhibited by aphidicolin. Here we focus on Panc-1 cells as a representative cell line that showed most significant effects of AZ10606120 on cell proliferation (Fig. 3) and the fastest migration among the PDAC cells (Additional file 6: Figure S5). In the first set of experiments we used a mechanical 96-pin wound maker. As shown in Fig. 8a-b, the inhibitor AZ10606120 (10 μM) did not show any effect on Panc-1 cells migration under these experimental conditions. We reasoned that the scratch procedure caused cells damage and ATP release that would obscure a possible effect of P2X7R on migration, because there might be other P2 receptors that contribute to cell migration as shown in studies on other cells [42, 43]. Therefore, we used another more gentle method, where the wound was created by removal of a silicone insert and the results are shown in Fig. 8c-d-e and Additional files 7 and 8. With this new protocol it was possible to detect that AZ10606120 caused a significant, about 30 % reduction in Panc-1 migration compared to the control.

**Fig. 9:**
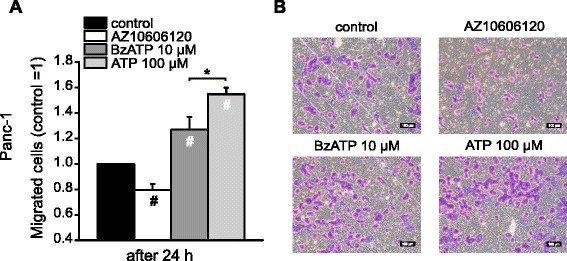
Effect of AZ10606120, BzATP and ATP on Panc-1 cells invasion. **a**. Difference in Panc-1 cells invasion. Cells were treated with or without AZ10606120 (10 μM), BzATP (10 μM) or ATP (100 μM). The PET membrane, 8.0 μm pore size, was coated with Geltrex Matrix. The number of migrated cells after 24 h was normalized with the control. **b**. Representative images of migrated cells after 24 h, fixed in cold methanol and stained with Crystal violet. All bars are 100 μm. The graph shows data from four experiments; each run was carried out in duplicates (mean ± SEM). Significant differences P < 0.05 (*, #) from the control (#) and between BzATP and ATP (*) are indicated

In order to evaluate whether the effect of P2X7R on PDAC cells migration was associated with invasion, i.e. the ability to degrade the ECM, a classical trans-well invasion assay was performed with Panc-1 cells (Fig. 9). Cells were incubated for 24 h with AZ10606120 (10 μM), BzATP (10 μM) and ATP (100 μM). Results in Fig. 9 show that the inhibitor caused about 20 % reduction in Panc-1 cell invasion compared to the control. BzATP and ATP stimulation increased cell migration by about 27 % and 55 %, respectively.

## Discussion

This study shows that pancreatic ductal adenocarcinoma cells express P2X7 receptors, which regulate PDAC cell behavior with respect to cell proliferation and cell death, as well as in cell migration and invasion.

P2X7R is expressed in pancreatic cells of ductal origin at the mRNA and protein levels (Figs. 1 and 2). Interestingly, rodent pancreatic acinar cells do not seem to express mRNA for P2X7R [30], although recent studies in pancreatic cancer field implicate transdifferentiation of acinar cells to duct cells as an important process in PDAC development [44]. In pancreatic duct cells, mRNA for P2X7R has a lower expression in all PDAC cell lines compared to HPDE cells (Fig. 1a), although we cannot be absolutely sure that HPDE cells are a faithful representative of healthy pancreatic ducts. Similar lower expression of receptor transcripts was found in uterine epithelial cancer compared to healthy controls [45]. In contrast, a number of other cancers types show increased mRNA levels for P2X7R compared to the normal tissues [46, 47]. Importantly, we find that the P2X7RA protein level was higher in PDAC cell lines compared HPDE (Fig. 1b). The discrepancy between mRNA and protein levels could be due to the fact that we detected transcripts from several isoforms (as it is impossible to design qPCR primers specific only for the isoform A), but we quantified the protein levels of the most easily detectable and studied isoform A (and B).

Although PDAC cells express other P2X and P2Y receptors (Additional file 1: Figure S1), we have several lines of evidence to demonstrate remarkable functionality of the P2X7 receptor. Stimulation with increasing exogenous ATP concentrations resulted in a reduction of BrdU incorporations in all cell lines (Figs. 3 and 5), and similar results were also obtained with stimulation with BzATP (Fig. 4). BzATP is considered as the most potent agonist for P2X7 receptors, although we cannot exclude contributions from other P2 receptors, which are activated at lower ATP concentration, or that BzATP activates potentially other receptors (e.g. P2X1 and P2X4 [48]). Nevertheless, we interpret these data to indicate the classical role of P2X7 receptor in cell death induced by high ATP concentrations and will return to this topic in more detail below. An additional argument for P2X7R functionality is the effect of two specific receptor inhibitors, AZ10606120 and A438079, which had different effects on PDAC behavior.

One of the best documented roles of P2X7R is pore formation and apoptosis/cell death [49]. We found that high concentrations of ATP caused pore formation in PDAC cell lines, as assayed by YoPro-1 uptake (Fig. 7). The competitive antagonist A438079, which is known to inhibit pore formation, decreased YoPro-1 uptake in all PDAC cell lines tested. This agrees with the P2X7 receptor-pore inhibitor characteristic of A438079 also described in pancreatic stellate cells, HEK293 cells and A375 human melanoma cells [17, 50, 51]. Although the pore formation is modest with high concentrations of ATP, as detected by the standard YoPro-1 analysis over 60 min, during long-term incubation with ATP, PDAC cells died with signatures of necrosis (Fig. 6). This was also reported for mesangial and SN4741 dopaminergic cells [13, 14] . Interestingly, although the pore-inhibitor A438079 had very modest effects on pore formation detected over a short period of incubation, it protected the cells from cell death during long-term incubation with high ATP concentrations (Fig. 5). Similar protective effects of the inhibitor in cell survival was detected in pancreatic stellate cells [17].

Let us now focus on the effect of the allosteric inhibitor, which, firstly, substantiated our arguments for functionality of P2X7R, and, second, revealed the proliferative effects of P2X7R. Thus, the specific allosteric inhibitor of P2X7R, AZ10606120 [52, 53], significantly reduced BrdU incorporation in PDAC cells (Fig. 3), which was not due to off-target cytotoxic effects (Additional file 4: Figure S3). Rather, we propose that the inhibitor decreased cell proliferation and thus revealed the proliferative potential of the P2X7 receptor over-expression in PDAC, confirming its pro-cancerous role. In “control” HPDE cells, which express lower protein levels of isoform A, the inhibitor had no effect with exogenous ATP addition. Notably in all cell lines, AZ10606120 had inhibitory effects even without added ATP, indicating that the over-expression and basal or tonic activation of P2X7R contribute to the hyperproliferative state of PDAC cells. Our findings that P2X7R is not only involved in cell death, but also in proliferation of cancer cells, are supported by studies on pancreatic stellate cells [17], on ovarian carcinoma cells [41] and also *in vivo* melanoma and colorectal tumor growth [27]. Importantly, some studies already suggest that AZ10606120 can be used to abolish the proliferative phenotype of P2X7R and reduce tumor size induced by melanoma B16 cells [27].

The two inhibitors A438079 and AZ10606120 had seemingly opposite effects on BrdU incorporation in PDAC cells, one being protective and the other being detrimental. This helped to reveal the double role of P2X7R—in cell proliferation and cell death, both of which would occur in a population of cells. It is not clarified at this stage what molecular mechanisms and/or molecular partners interacting with the receptor could explain these seemingly disparate effects on cell survival.

In addition to cell proliferation, cancer cell migration and invasion are important in cancer behaviour. Our study shows that P2X7R is important in these two cancer hallmarks as the allosteric modulator AZ10606120 significantly reduces both PDAC cell migration (Fig. 8, Additional files 7 and 8) and invasion (Fig. 9). P2X7R also contributes to cancer cell migration and invasion as demonstrated in other types of cancer [47], though also other P2 receptors (e.g. P2Y2) contribute to cancer cell migration and invasion [54–56].

Taken together, our results show that multiple functions of PDAC cell lines are regulated by P2X7R: cell proliferation, cell migration and invasion, pore-formation and cell death. In a complex tumor, cells can encounter sites of lower and high ATP concentrations, e.g. at the periphery of tumor and at the core necrotic part of the tumor, respectively. Our results are in line with the model presented by Roger *et al.* [57] and with the “run or die” hypothesis in cancer, [6], in which cancer cells have a pro-cancerous basal activity of P2X7R that leads to proliferation and migration/invasion. Sustained activation, with high concentrations of exogenous ATP/BzATP, in a closed system (like a microplate well), which can reproduce the cancer necrotic core, leads to cancer cell death. In contrast, in an open system (like a Boyden chamber), which can reproduce the edge of the tumor, cancer cells have the possibility to “move”. ATP can be read like a stress signal and consequently cancer cells can increase the P2X7-pro-invasive phenotype and escape the harmful cancer microenvironment and invade new places. However, further investigations are needed to clarify the role of P2X7R in a complex solid tumor microenvironment, such as that of pancreatic cancer, and importantly to explore the potential interaction between P2X7R expressing cells—PDAC, PSCs, and immune cells, and as a recent report also suggests, cancer stem cells [58]. In order to develop drug therapies, it will be important to resolve in which cell type the P2X7R-related events dominate cancer progression, as for example studied recently by Adinolfi et al. [59].

## Conclusions

In conclusion, our study shows that at least two P2X7 receptor isoforms are expressed in PDAC and contributes to the basic cancer cell behavior in proliferation as wells as to cell death, and to cell migration and invasion. Therefore, we propose that the receptor could be exploited as a potential therapeutic target for treatment of pancreatic cancer.

## Methods

### Cell cultures

Human pancreatic ductal adenocarcinoma cell lines were purchased from ATTC (Manassas, VA, USA). AsPC-1 (CRL-1682) and BxPC-3 (CRL-1687) were grown in RPMI-1640 medium, Capan-1 (HTB-79) in Iscove’s Modified DMEM (IMDM), Panc-1 (CRL-1469) in Dulbecco’s Modified Eagles Medium (DMEM) and MiaPaCa-2 (CRL-1420) in DMEM/Ham’s F12. Cell culture media contained stable glutamine, 10 % (20 % for Capan-1) fetal bovine serum (FBS) (PAA Laboratories; A15-151 Gold) plus 2.5 % of horse serum for MiaPaCa-2 (Biochrom), 100 U/ml penicillin and 100 μg/ml streptomycin. The human pancreatic duct epithelial cell line HPDE6-E6E7 (H6c7) [60, 61], which we refer to as HPDE, was obtained from Dr. Ming-Sound Tsao. HPDE cells were grown in KBM Basal Medium (Lonza, CC-3101), which is part of the KGM-Bullet Kit (Lonza, CC-3111). Cells were grown at 37 °C in a humidified atmosphere with 5 % CO_2_. All standard chemicals were purchased from Sigma-Aldrich unless otherwise stated.

### RNA isolation, RT-PCR and real time PCR

Cells were cultured to confluence in a Petri dish and then RNA was isolated with RNeasy Mini Kit (Qiagen) according to the manufacturer’s instructions. DNase I, Qiagen was used. The cDNA was synthesized using the RevertAid First Strand cDNA synthesis kit (Fermentas). For RT-PCR cDNA, Jumpstart Taq DNA, primers and dNTP mix were used; the amplification parameters were as follows: one cycle at 95 °C for 1 min, 45 cycles at 95 °C for 20 s, 57 °C for 30 s, 72 °C for 1.15 min, one final cycle at 72 °C for 5 min. The transcripts were run electrophoretically in 1.2 % agarose gel. The real time PCR reactions were run using Roche FastStart Universal SYBR Green Master (ROX) with parameters as follows: one cycle at 50 °C for 2 min and one cycle at 95 °C for 10 min followed by 40 amplification cycles at 95 °C for 15 s, 57 °C for 1 min, 72 °C for 1 min and at the end a dissociation step at 95 °C for 15 s, 60 °C for 15 s, 95 °C for 15 s. Data were evaluated with SDS 2.4 software (Applied Biosystems). Primers were designed using Primer-BLAST (NCBI) and synthesized by TAG Copenhagen A/S, DK. Three housekeeping genes that were found quite stable in normal and cancerous pancreatic tissues [62] were used for normalization: *β-actin*, β-glucuronidase (*GUSB*) and glutaminyl-tRNA synthetase (*QARS*). The relative amount of the target mRNA was calculated from a standard curve constructed from the results of a serial dilution run on each plate, using Pfaffl method [63].

### Western blot

Cells were cultured to confluence in a Petri dish and lysed by adding 5x diluted lysis buffer (50 mM Tris-base, 0.25 M NaCl, 10 mM EDTA, 0.5 % NP40, 1 % TritonX-100, 4 mM NaF, pH 7.5) with 1x protease inhibitor. The final lysate was centrifuged at 15,000 g at 4 °C for 15 min. Protein concentration was estimated with a Coomassie protein assay. Protein samples were reduced by heating at 98 °C for 10 min with 50 mM DTT. 50 μg of proteins were loaded and run in 10 % polyacrylamide precast gels (Invitrogen) and blotted to PVDF membranes (Invitrogen). Membranes were blocked with 5 % skim milk solution in TBS-Tween 0.1 % buffer for 1 h at room temperature and incubated overnight at 4 °C with primary antibodies against P2X7R extracellular loop (1:200 rabbit polyclonal, Alomone, APR-008) and β-Actin (1:1000 mouse monoclonal C4, Santa Cruz, Sc-47778). Blots were then incubated with appropriate HRP-conjugated antibodies and developed with EZ-ECL (Biological Industries) and visualized on Fusion FX (Vilber Lourmat) and band intensity was calculated using Bio1D software.

### Confocal fluorescence microscopy

Cells were grown on glass coverslip, and then fixed in 4 % paraformaldehyde for 15 min at room temperature. Cells were treated with 0.1 M TRIS-glycine (pH 7.4) for 15 min; permeabilized 0.3 % Tween-20 and blocked with 5 % BSA for 30 min. Cells were incubated with antibody against P2X7R extracellular loop (1:100 rabbit polyclonal, Alomone, APR-008) overnight at 4 °C. Then preparations were incubated with a secondary antibody conjugated to Alexa 568. DAPI (Molecular Probes) was used for nuclear staining. Fluorescence was examined with 40x 1.3 NA objective in Leica SP 5X MP confocal laser scanning microscope. Images and overlays were analyzed in Leica LAS AF software.

### Cell proliferation

Cells were plated in white, clear bottom 96-well plates. ATP and 2′-3′-O-(4-benzoylbenzoyl)-ATP (BzATP) were dissolved in media and the pH was adjusted to 7.4 with NaOH. The indicated concentrations of ATP or BzATP were added with and without P2X7R inhibitors (10 μM AZ 10606120, 10 μM A438079 and 100 nM KN-62, Tocris) in 1 % serum media (0 % of serum for the experiments with low ATP concentrations—from 0.5 μM to 10 μM). After 60 h of incubation at 37 °C in a humidified atmosphere with 5 % CO_2_, cells were treated according to the manufacturer’s instruction of the Roche Cell Proliferation ELISA, BrdU chemiluminescence kit. Luminescence was read in FLUOstar OPTIMA (BMG Labtech).

### Cell viability assays

To check the possible cytotoxicity of the inhibitor AZ10606120 (10 μM), the cell viability, was determined using Trypan blue exclusion. Panc-1 and BxPC-3 cells were grown in 6-well plates in 1 % serum. After 20 h, 40 h and 60 h of incubation at 37 °C in a humidified atmosphere with 5 % CO_2_, cells were incubated 10 min with Trypan blue. Bright field images were taken with Leica DMI6000B microscope and 10x objective. In order to determine the mode of two common cell deaths (apoptosis, necrosis) two assays were used. For apoptosis, Panc-1 cells were plated in white 96-well plates with clear bottom. ATP solution adjusted to pH 7.4 was added in 1 % serum media for a final concentration of 1 mM. Apoptosis inducer AT101 [64] was used as a control. After 20 h of incubation, caspase 3/7 activation was determined. Cells were treated according to the manufacturer’s instruction of the Promega Caspase-Glo® 3/7 Assay. Luminescence was read by FLUOstar OPTIMA. For necrosis assay Panc-1 cells were grown in 35 mm glass-bottom dishes, after 20 h of treatment with ATP cells were incubated for 15 min with Annexin V-488 and Propidium Iodide (PI) (Life Technologies). Pictures were taken with 40x 1.3 NA objective in Leica SP 5X MP confocal laser scanning microscope, CLSM. Images and overlays were analyzed in Leica LAS AF software.

### Pore formation

Cells were plated in black 96-well plates with clear bottom. When a confluence around 80–90 % was reached, the media was changed to a physiological buffer (140 mM NaCl, 10 mM HEPES, 1 mM MgCl_2_, 0.4 mM KH_2_PO_4_, 1.6 mM K_2_HPO_4_, 1.5 mM CaCl_2_) with 11.1 mM of glucose for AsPc-1 and BxPC-3 and 25 mM for Panc-1. Cells were incubated with AZ10606120 (10 μM) or A438079 (10 μM) for 1 h. Then cells were incubated with Yo-Pro-1 Iodide (2.5 μM, Molecular Probes) for 15 min. ATP was dissolved in a physiological buffer and the indicated concentrations were added. Ionomycin (5 μM) was added at the end of each experiment. Fluorescence was read every 10 min by FLUOstar OPTIMA.

### Wound healing assay

The scratch assay was performed in two different methods. In the first method, cells were grown to confluence in 96-well Essen ImageLock plate, and then incubated with 10 μM AZ10606120 for 1 h and 5 μM aphidicolin (to stop proliferation). The wounds were made simultaneously in all wells with a 96-pin WoundMaker (Essen BioScience), washed with PBS and phase contrast pictures were taken every 2 h for 24 h in the IncuCyte ZOOM Kinetic Imaging System. Pictures were processed with the IncuCyte software using the relative wound density *(%RWD*) option, which measures the density of the wound with respect to the density of the cell region. The program uses the following formula: *%RWD = 100 × w(t) − w(0)/c(t) − w(t)*; where, *w(t)* is the density of wounded area at time *t* and *c(t)* is the density of cell region at time *t*. To avoid cells damage, a second method was used. Ibidi biocompatible silicone culture-insert was stuck in the center of a 35 mm Petri dish and cells were plated in the same number in the two chambers. When the confluence was reached, the insert was removed and afterwards media with and without AZ10606120 (10 μM) was added. 10x phase contrast pictures were taken every 1 h for 24 h in the Nikon BioStation IM-Q that allowed a temperature- and gas-controlled environment. Images were analyzed by Nikon NIS-Elements AR 4.13.03 software.

### Invasion assay

The inserts for 24-well plates (transparent PET membrane, 8.0 μm pore size, Falcon) were coated with Geltrex Matrix (Invitrogen). 30.000 Panc-1 cells with aphidicolin (5 μM) and with/without AZ10606120 (10 μM), BzATP (10 μM) and ATP (100 μM) were placed in the upper chamber in 1 % of serum, and media with 10 % of serum was added in the lower chamber. After 24 h incubation at 37 °C in a humidified atmosphere with 5 % CO_2_, cells were fixed in cold methanol and stained with Crystal Violet. Bright field images were taken with 10x objective in Leica DMI6000B microscope. Cells were counted by ImageJ, NIH.

### Chemicals and statistics

All chemicals/kits were purchased from Sigma-Aldrich unless otherwise stated. Data are shown as mean values ± standard error of mean (SEM) and *n* represents the number of biological replicates. Statistical analysis on data was performed by using Students *t*-test and One-way Analysis of Variance (ANOVA) with Holm-Sidak method using SigmaPlot 11.0. Data were analyzed in Origin or Microsoft Excel.

## Additional files

Additional file 1: Figure S1. Expression of P2X7R in PDACs and HPDE cells. Real time PCR analysis of P2X and P2Y receptors expression in HPDE and PDAC cells. The data were normalized with respect to the three housekeeping genes: *β-actin*, β glucuronidase (*GUSB*) and glutaminyl-tRNA synthetase (*QARS*) and expression in HPDE was set to be 1. The bargraph shows data for four experiments (mean ± SEM). The relative amount of mRNA was calculated from a standard curve run on each plate. Significant difference of expression in PDAC in comparison to HPDE cells is indicated P < 0.05 (*) and P < 0.001 (**). (PDF 201 kb) Additional file 2: Table S1. Primers used for RT-PCR and Real Time PCR on PDACs and HPDE (DOC 33 kb) Additional file 3: Figure S2. Effect of low ATP concentrations on BrdU incorporation in Panc-1 cells. The graph shows the effect of low concentrations of added (exogenous) ATP (0.5 μM, 3 μM, 7 μM and 10 μM), or control (no exogenous ATP added), on BrdU incorporation in Panc-1 cell line after 60 h. The results were normalized to 0 % serum control (100). The graph shows data from four independent experiments (mean ± SEM); where each run was carried out in triplicates. No significant differences compared to the control were detected. (PDF 59 kb) Additional file 4: Figure S3. Absence of cytotoxicity of AZ10606120 on PDAC cells. Representative bright field images of A. Panc-1 and B. BxPC-3 at 20, 40 and 60 h incubation with AZ10606120 (10 μM) and control. Cells were stained with Trypan Blue at the time points given above. In every experiment pictures were taken from eight to twelve fields (*n* = 3). All bars are 50 μm. (PDF 1214 kb) Additional file 5: Figure S4. Pore formation with 1 mM ATP. Yo-Pro-1 (2.5 μM) uptake was monitored after stimulation 1 mM ATP alone, and in combination with a pre-inhibition (1 h) with AZ10606120 (10 μM) or A438079 (10 μM). Fluorescence of YoPro-1 (Ex. 490 nm/Em. 510 nm) was measured in Panc-1 (**A**, **B**), AsPC-1 (**C**, **D**) and BxPC-3 (**E**, **F**). **A**, **C**, **E**. Fluorescence values taken every 10 min for 60 min and normalized to the average of values before administration of ATP. **B**, **D**, **F**. Values of normalized fluorescence at 60 min. The lines and bars-graphs show data of four experiments (mean ± SEM); each run was carried out in triplicates. Significant differences P < 0.05 (*, #) and P < 0.001 (##) from the respective control (#, ##) and with/without inhibitor (*) are indicated. (PDF 551 kb) Additional file 6: Figure S5. PDAC cells migration. Confluent Panc-1, BxPC-3, AsPC-3 and MiaPaCa-2 cells, grown in 96-well plate were scratched with a 96-pin wound maker. The graph shows the percentage of closed wound. Pictures were taken every 2 h. Images were analyzed with the IncuCyte software using the relative wound density option, which measures the density of the wound according to the density of the cell region. The graphs show data from three to six experiments (mean ± SEM); each run was carried out in triplicates. Significant differences among cell lines P < 0.05 (*) and P < 0.001 (**) are calculated on the slope of the curves. (PDF 352 kb) Additional file 7:
**Video S6.** Migration of Panc-1 cells grown in the two chambers of an Ibidi silicon insert. Phase contrast pictures were taken every hour for 24 h. (MP4 3335 kb) Additional file 8:
**Video S7.** Migration of Panc-1 cells in presence of AZ10606120 inhibitor. Phase contrast pictures were taken every hour for 24 h. (MP4 2735 kb)
