# Supplementary figures and images for: The P2X7 receptor regulates cell survival, migration and invasion of pancreatic ductal adenocarcinoma cells
Source: Mol Cancer. 2015 Nov 25;14:203. doi: 10.1186/s12943-015-0472-4 (PMC4660609; doi:10.1186/s12943-015-0472-4)

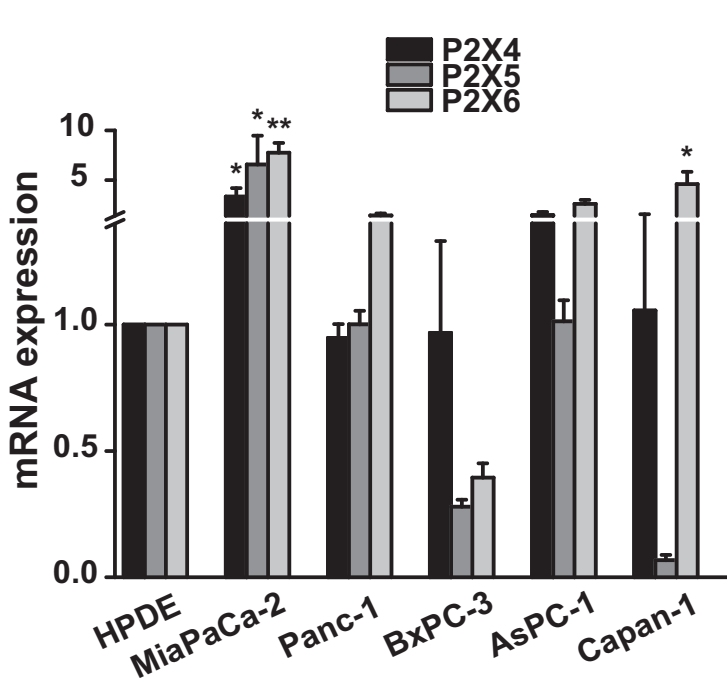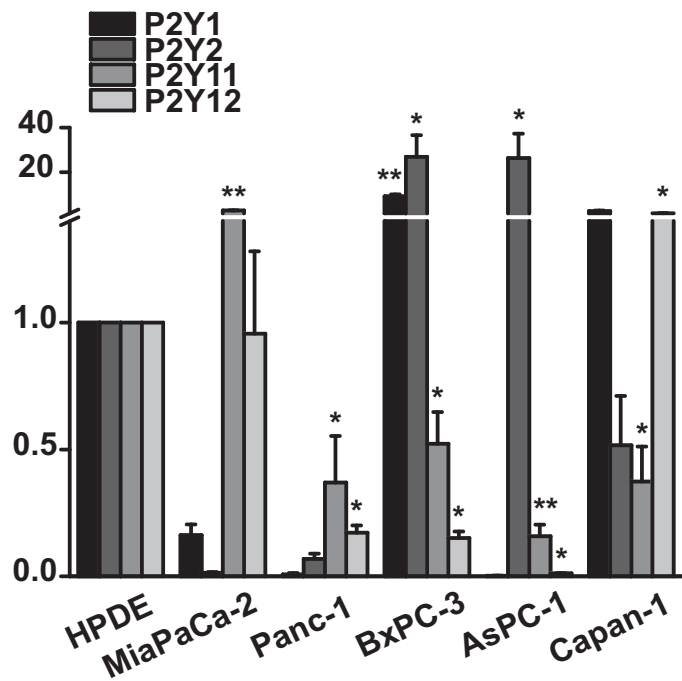

Supplement: Additional file 1: Figure S1. — Expression of P2X7R in PDACs and HPDE cells. Real time PCR analysis of P2X and P2Y receptors expression in HPDE and PDAC cells. The data were normalized with respect to the three housekeeping genes: β-actin, β glucuronidase (GUSB) and glutaminyl-tRNA synthetase (QARS) and expression in HPDE was set to be 1. The bargraph shows data for four experiments (mean ± SEM). The relative amount of mRNA was calculated from a standard curve run on each plate. Significant difference of expression in PDAC in comparison to HPDE cells is indicated P < 0.05 (*) and P < 0.001 (**). (PDF 201 kb) [file 12943_2015_472_MOESM1_ESM.pdf]

# Panc-1

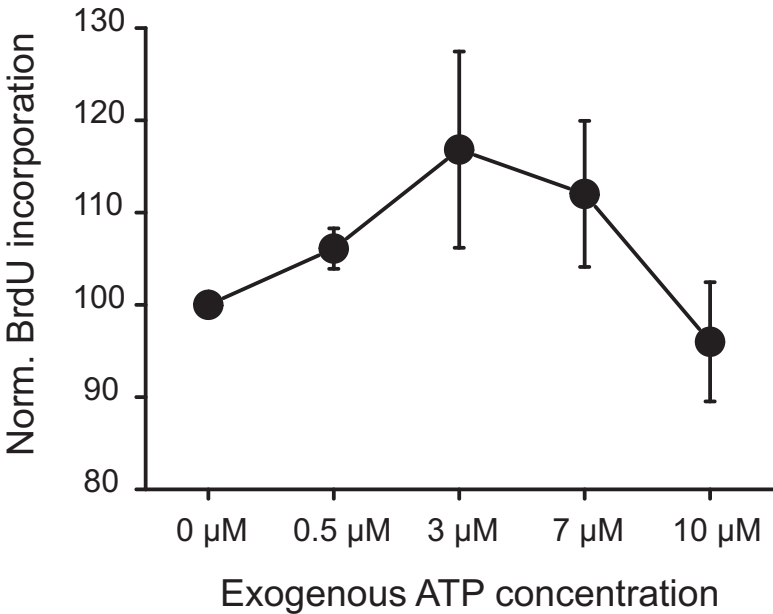

Supplement: Additional file 3: Figure S2. — Effect of low ATP concentrations on BrdU incorporation in Panc-1 cells. The graph shows the effect of low concentrations of added (exogenous) ATP (0.5 μM, 3 μM, 7 μM and 10 μM), or control (no exogenous ATP added), on BrdU incorporation in Panc-1 cell line after 60 h. The results were normalized to 0 % serum control (100). The graph shows data from four independent experiments (mean ± SEM); where each run was carried out in triplicates. No significant differences compared to the control were detected. (PDF 59 kb) [file 12943_2015_472_MOESM3_ESM.pdf]

## Panc-1

**A**

20 h

40 h

60 h

control

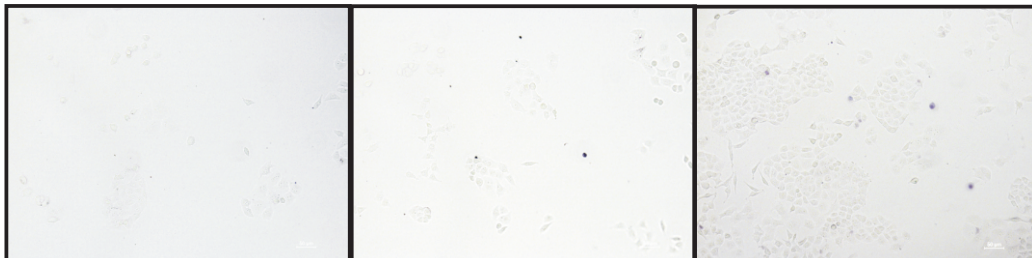

AZ10606120

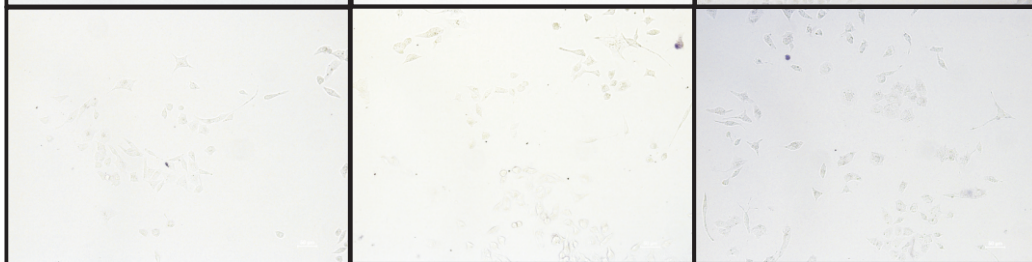

## BxPC-3

**B**

20 h

40 h

60 h

control

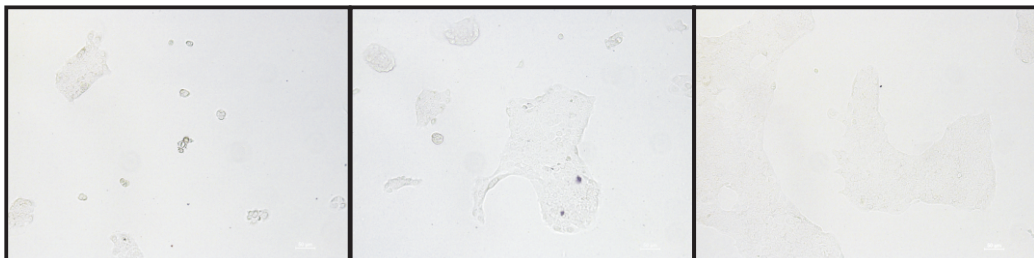

AZ10606120

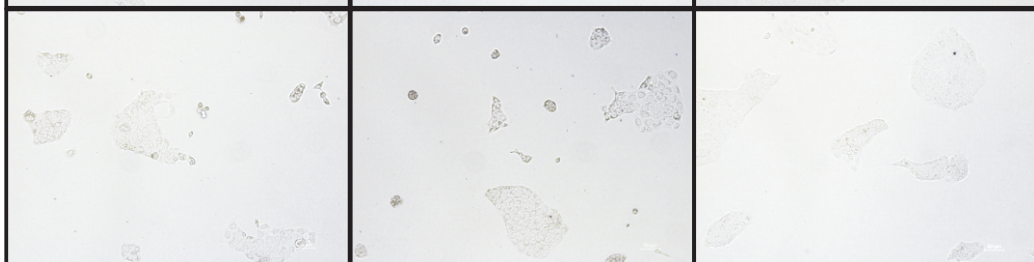

Supplement: Additional file 4: Figure S3. — Absence of cytotoxicity of AZ10606120 on PDAC cells. Representative bright field images of A. Panc-1 and B. BxPC-3 at 20, 40 and 60 h incubation with AZ10606120 (10 μM) and control. Cells were stained with Trypan Blue at the time points given above. In every experiment pictures were taken from eight to twelve fields (n = 3). All bars are 50 μm. (PDF 1214 kb) [file 12943_2015_472_MOESM4_ESM.pdf]

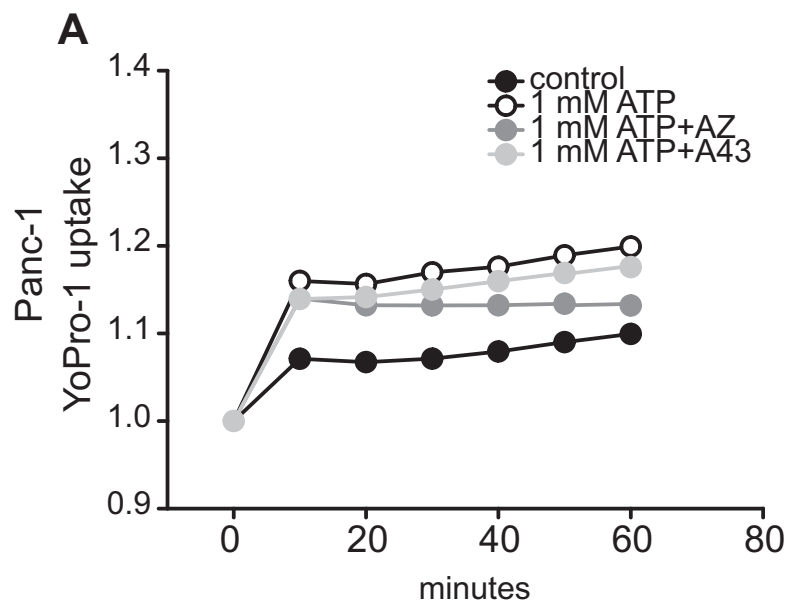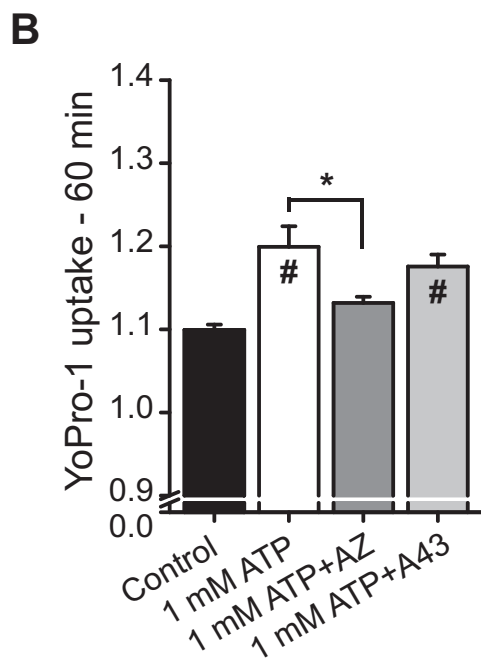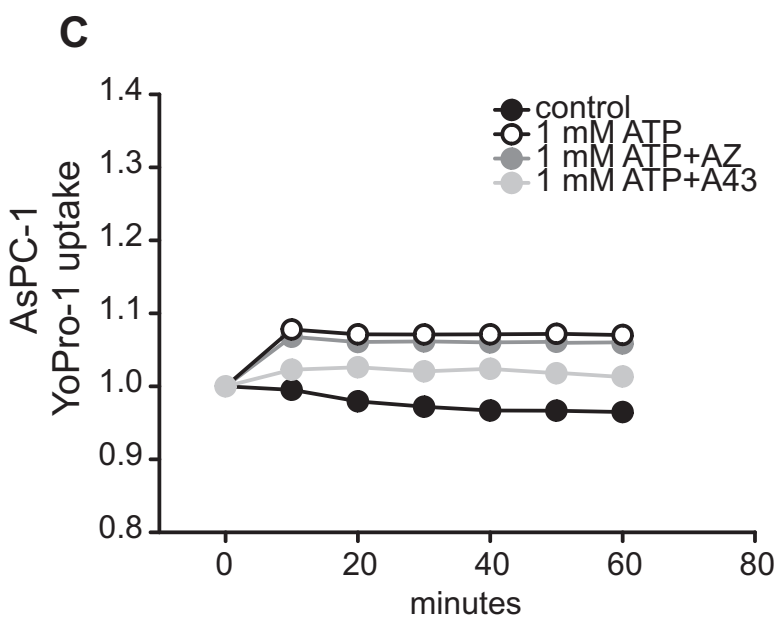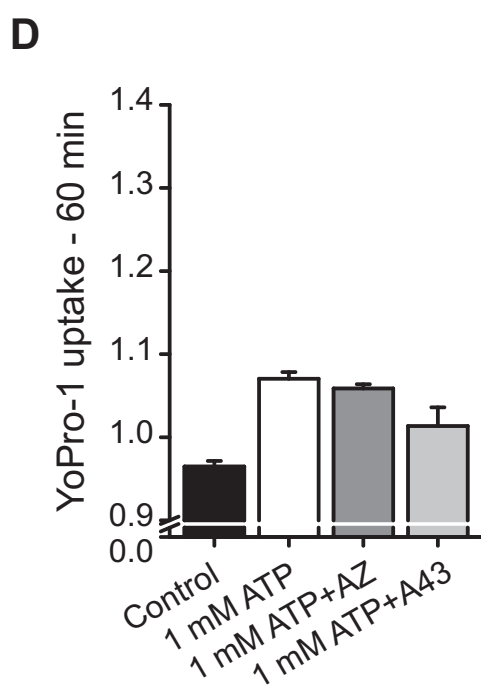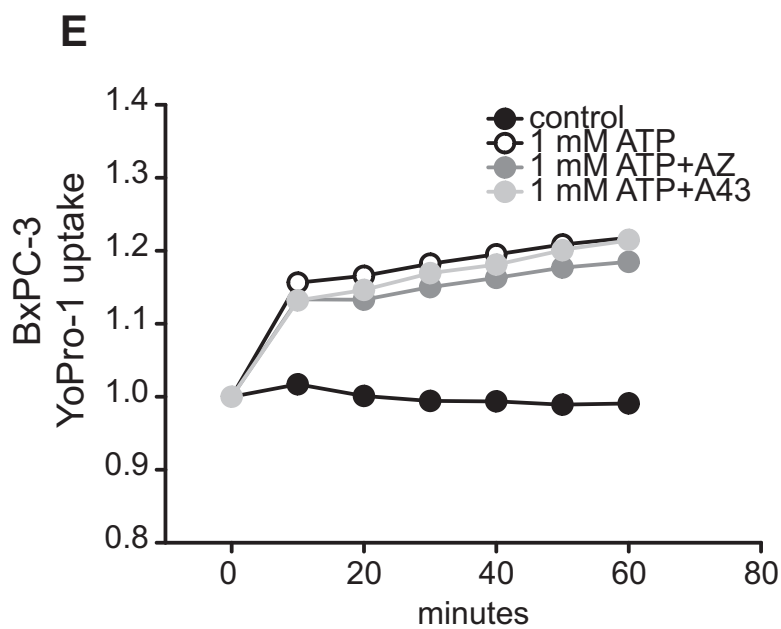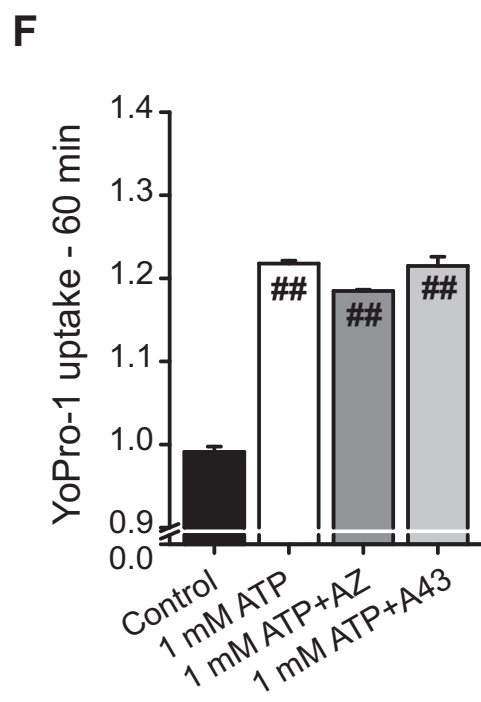

Supplement: Additional file 5: Figure S4. — Pore formation with 1 mM ATP. Yo-Pro-1 (2.5 μM) uptake was monitored after stimulation 1 mM ATP alone, and in combination with a pre-inhibition (1 h) with AZ10606120 (10 μM) or A438079 (10 μM). Fluorescence of YoPro-1 (Ex. 490 nm/Em. 510 nm) was measured in Panc-1 (A, B), AsPC-1 (C, D) and BxPC-3 (E, F). A, C, E. Fluorescence values taken every 10 min for 60 min and normalized to the average of values before administration of ATP. B, D, F. Values of normalized fluorescence at 60 min. The lines and bars-graphs show data of four experiments (mean ± SEM); each run was carried out in triplicates. Significant differences P < 0.05 (*, #) and P < 0.001 (##) from the respective control (#, ##) and with/without inhibitor (*) are indicated. (PDF 551 kb) [file 12943_2015_472_MOESM5_ESM.pdf]

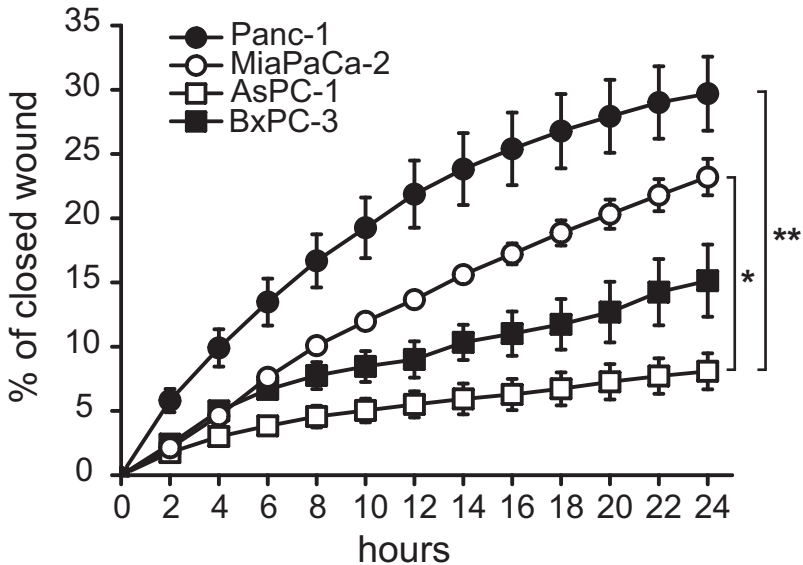

Supplement: Additional file 6: Figure S5. — PDAC cells migration. Confluent Panc-1, BxPC-3, AsPC-3 and MiaPaCa-2 cells, grown in 96-well plate were scratched with a 96-pin wound maker. The graph shows the percentage of closed wound. Pictures were taken every 2 h. Images were analyzed with the IncuCyte software using the relative wound density option, which measures the density of the wound according to the density of the cell region. The graphs show data from three to six experiments (mean ± SEM); each run was carried out in triplicates. Significant differences among cell lines P < 0.05 (*) and P < 0.001 (**) are calculated on the slope of the curves. (PDF 352 kb) [file 12943_2015_472_MOESM6_ESM.pdf]
