# Supplementary material for: The P2X7 receptor regulates cell survival, migration and invasion of pancreatic ductal adenocarcinoma cells
Source: Mol Cancer. 2015 Nov 25;14:203. doi: 10.1186/s12943-015-0472-4 (PMC4660609; doi:10.1186/s12943-015-0472-4)
Supplement: Additional file 2: Table S1. — Primers used for RT-PCR and Real Time PCR on PDACs and HPDE (DOC 33 kb) [file 12943_2015_472_MOESM2_ESM.doc]

Additional file 2: Table S1. Primers used for RT-PCR and Real Time PCR on PDACs and HPDE

| **Primers** | **Accession Numbers** | **Sequence** | **Product length** |
| --- | --- | --- | --- |
| **P2X4 FW** | [GenBank: BC033826.1] | GAGATTCCAGATGCGACCACT | 112 bp |
| **P2X4 RW** |  | ACCCGTTGAAAGCTACGCAC |  |
| **P2X5 FW** | [GenBank: AF016709.1] | CTGTCGCTGTTCGACTACAAG | 112 bp |
| **P2X5 RW** |  | CCCATACGACCAGGTACGC |  |
| **P2X6 FW** | [GenBank: AF065385.1] | TGCAGTTTGGGATCGTGGTC | 195 bp |
| **P2X6 RW** |  | ACACGTTCTCTCCCTGAGGT |  |
| **P2Y1 FW** | [GenBank: BC074785.2] | AATGCGATCTGTATCAGCGTG | 118 bp |
| **P2Y1 RW** |  | TGGTGTCGTAACAGGTGATGG |  |
| **P2Y2 FW** | [GenBank: BC028135.1] | CCGCTTCAACGAGGACTTCAA | 211 bp |
| **P2Y2 RW** |  | GCGGGCGTAGTAATAGACCA |  |
| **P2Y11 FW** | [GenBank: AF030335.1] | TTGAACCTCAGGAGGGTTGTG | 214 bp |
| **P2Y11 RW** |  | ACTTGGCACCCGAGACGTT |  |
| **P2Y12 FW** | [GenBank: BC017898.1] | CACTGCTCTACACTGTCCTGT | 190 bp |
| **P2Y12 RW** |  | AGTGGTCCTGTTCCCAGTTTG |  |
